# Protective Effects of *Fructus mume* Extract Against Deoxynivalenol-Induced Intestinal and Liver Injury in Mice

**DOI:** 10.3390/biology15141172

**Published:** 2026-07-16

**Authors:** Jiali Liu, Shipeng Liu, Xiaowei Zhou, Zhaoran Zhong, Qingyue Hu, Qinjin Li, Zhaoyan Lin, Xiaohong Huang, Bohan Zheng

**Affiliations:** 1 College of Animal Science, Fujian Agriculture and Forestry University, Fuzhou 350002, China; liujiali@fafu.edu.cn (J.L.); corhgth@163.com (S.L.); zhongzhaoran@fafu.edu.cn (Z.Z.); hqycmm@163.com (Q.H.); liqinjin0809@163.com (Q.L.); lynn_1209@fafu.edu.cn (Z.L.); 2 University Key Laboratory for Integrated Chinese Traditional and Western Veterinary Medicine and Animal Healthcare in Fujian Province/Fujian Key Laboratory of Traditional Chinese Veterinary Medicine and Animal Health, Fujian Agriculture and Forestry University, Fuzhou 350002, China; zxwdyx1206@163.com; 3 Institute of Oceanology, Fujian Agriculture and Forestry University, Fuzhou 350002, China

**Keywords:** *Fructus mume* extract, deoxynivalenol, *Fusarium*, intestinal injury, oxidative stress, inflammation, gut–liver axis

## Abstract

Deoxynivalenol (DON), a prevalent mycotoxin, induces intestinal and hepatic injury. *Fructus mume* extract (FME) possesses bioactive properties, yet its protective role against DON remains unclear. This study aimed to investigate the protective mechanisms of FME in DON-challenged mice. Male C57BL/6 mice were divided into control, DON (3 mg/kg), and DON with low-, medium-, or high-dose FME groups for 4 weeks. Analyses included histopathology, UPLC-Q-TOF-MS, network pharmacology, biochemistry, qRT-PCR, immunohistochemistry, a TUNEL assay, metagenomics, and metabolomics. FME significantly alleviated growth inhibition and tissue damage. Among the 38 components identified by UPLC-Q-TOF-MS, all 38 acted on 156 genes, including *IL-1β*, *caspase3*, and *BAX*, to alleviate DON-induced intestinal and hepatic injury. FME enhanced hepatic antioxidant capacity and reduced inflammation by suppressing *NF-κB* signaling. FME upregulated tight junction proteins, inhibited apoptosis, and restored microbial diversity by enriching beneficial bacteria. Metabolomics revealed FME reversed DON-induced metabolic disruptions in the liver. Correlation analysis indicated FME remodeled the microbiota–liver metabolite network. In conclusion, FME attenuates DON-induced intestinal injury by modulating the gut–liver axis through antioxidant, anti-inflammatory, and anti-apoptotic activities.

## Simple Summary

A common toxin found in grains and animal feed can damage the intestines and liver, threatening food safety and animal health. This study tested whether a natural extract from a traditional Chinese fruit, *Fructus mume*, could reduce such damage. Using a mouse model, we found that the extract significantly relieved intestinal and liver injury. It reduced inflammation, repaired the gut barrier, increased beneficial gut bacteria, and restored normal liver metabolism. These effects worked together to protect the animals from toxin-induced harm. Our findings suggest that *Fructus mume* extract is a promising natural strategy to counter the harmful effects of this toxin. This research provides scientific support for developing safer animal feed and greener feed additives, ultimately benefiting food safety and animal health.

## 1. Introduction

The intestine serves as the primary digestive, absorptive, and immune organ in the body [1]. Its structural integrity and functional homeostasis directly determine host health, and it is also the primary target organ for exogenous toxins [2,3]. Deoxynivalenol (DON), also known as vomitoxin, is a type B trichothecene mycotoxin primarily produced by *Fusarium graminearum*, as well as by other *Fusarium* species such as *F. culmorum* and *F. poae*. It widely contaminates grains and feeds, including wheat, corn, and barley, making it one of the most prevalent and hazardous mycotoxins worldwide [4]. Owing to its high thermal stability and acid resistance, DON cannot be completely degraded by conventional food processing procedures [5,6]. Long-term dietary or feed-borne exposure poses severe threats to human and animal health and has become a critical issue that restricts food safety and the breeding of healthy livestock [7].

The toxic effects of DON are extensive, among which intestinal injury is the most direct and prominent [8]. As a potent cytotoxic agent, DON specifically targets highly proliferative intestinal mucosal epithelial cells [9]. It impairs protein synthesis, induces oxidative stress and inflammatory responses, disrupts tight junction structures, and consequently damages the intestinal physical, chemical, biological, and immune barriers [10,11]. Studies have confirmed that DON exposure causes intestinal mucosal atrophy, villus disruption, crypt hyperplasia, and increased intestinal permeability in mice, accompanied by gut microbiota dysbiosis and excessive release of pro-inflammatory cytokines, leading to characteristic toxic symptoms such as diarrhea, vomiting, and anorexia [12]. In severe cases, DON triggers liver injury via the gut–liver axis, forming a cascade amplification of toxicity [13,14]. Furthermore, DON is classified as a Group 3 carcinogen, and long-term low-dose exposure may increase the risk of tumorigenesis, further aggravating its health hazards [15]. At present, safe and effective strategies for preventing and controlling DON-induced intestinal injury remain limited [11]. Therefore, exploring natural, efficient, and low-toxicity protective agents has become a research hotspot in this field.

Fructus mume (the dried near-ripe fruit of *Prunus mume* (Sieb.) Sieb. et Zucc., Rosaceae) is a traditional edible and medicinal herb in China which can be traced back to Shennong’s *Classic of Materia Medica* [16]. It has a long history of clinical use for treating chronic diarrhea and dysentery, vomiting, and abdominal pain due to ascariasis, and its traditional effect of “astringing the intestine to stop diarrhea” is closely associated with intestinal health [17]. Contemporary pharmacological research indicates that *Fructus mume* extract contains abundant organic acids, flavonoids, polysaccharides, polyphenols, and other active components, which endow it with multiple bioactivities, including intestinal function regulation, anti-inflammation, antioxidant activity, and antimicrobial effects [18,19,20]. FME improves intestinal function by inhibiting excessive smooth muscle contraction, regulating gastrointestinal hormones, promoting beneficial intestinal bacteria, and repairing the intestinal mucosal epithelial barrier [17,21]. Meanwhile, FME alleviates tissue inflammatory injury by scavenging free radicals, downregulating pro-inflammatory factors, and modulating inflammatory signaling pathways, thereby showing potential protective effects against intestinal injury induced by various stimuli [22]. In addition, FME can inhibit the growth of pathogenic bacteria by destroying their cell walls and interfering with their metabolic activities, thereby maintaining a stable intestinal microecological environment and providing double protection for intestinal health [23].

Current studies on FME mainly focus on its antimicrobial, hepatoprotective, gut microbiota-regulating, and heavy metal-detoxifying activities. However, its protective effect against DON-induced intestinal injury and the underlying molecular mechanisms remain largely unclear. This work aims to provide new ideas and experimental evidence for developing natural and safe agents to alleviate DON-induced injury, and to expand new directions for the thorough development and high-value utilization of *Fructus mume* as an edible-medicinal resource.

## 2. Materials and Methods

### 2.1. Chemicals, Reagents, and Extract

*Fructus mume* extract (FME) was purchased from Desifu Biotechnology Co., Ltd. (Nanjing, China), Item No.: DASF2643, Batch No.: 250120W. The extract was prepared through extraction with water at a solvent-to-material ratio of 10:1, at 90 °C, for 1 h per extraction, repeated three times. Deoxynivalenol (DON, C_15_H_20_O_6_, molecular weight = 296.31) with a purity of ≥90% was obtained from Yujing Technology Co., Ltd. (Shanghai, China).

### 2.2. Animals and Experimental Design

Male C57BL/6 mice (body weight 18 g, age 3–4 weeks) were obtained from Slack Laboratory Animal Co., Ltd., in Shanghai, China (production license No. SCXK (Hu) 2022-0004). This experiment was approved by the Ethics Committee of Fujian Agriculture and Forestry University, with the approval number PZCASFAFU26064. Mice were raised in a standard laboratory environment with a 12 h light/dark cycle, a temperature between 20 and 24 °C, and 45% relative humidity. Food and water were available at all times. Before use, all cages, corn cob bedding, and water bottles were disinfected. Bedding and cages were replaced and cleaned daily to maintain a hygienic environment.

**Figure 1:**
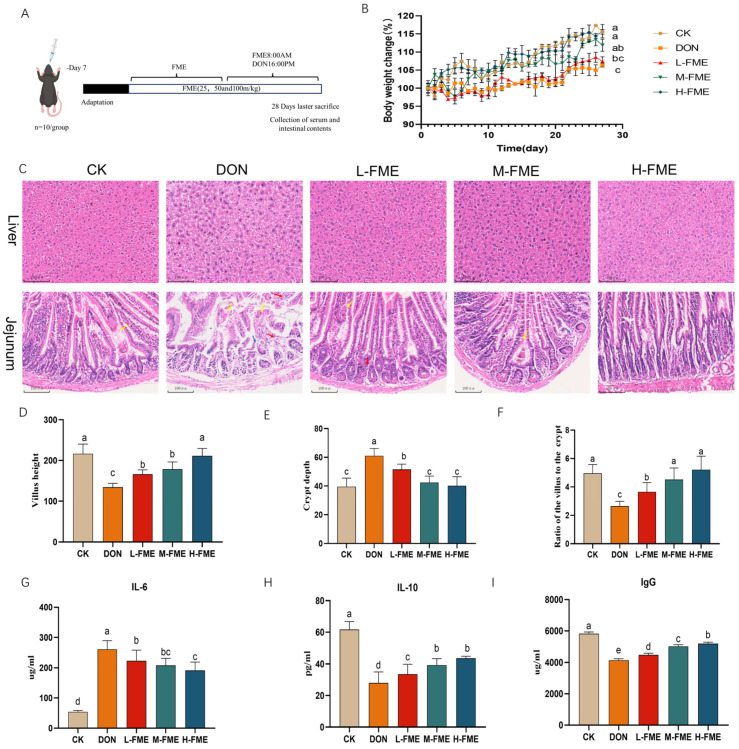
Effects of *Fructus mume* extract (FME) on growth performance, histopathology, and inflammatory-immune indices in deoxynivalenol (DON)-exposed mice. (**A**) Schematic diagram of the animal experimental design. (**B**) Rate of body weight change in mice over 28 days. (**C**) Hematoxylin and eosin (H&E) staining of liver and jejunum tissues. Representative histomorphological images of jejunum tissues (H&E staining, scale bar = 100 μm). Arrows of different colors indicate the following: red arrow—inflammatory cell infiltration; yellow arrow—villus atrophy and blunting; blue arrow—crypt destruction (scale bar: 100 μm; applies to all panels). (**D**) Statistical analysis of jejunal villus height. (**E**) Statistical analysis of jejunal crypt depth. (**F**) Statistical analysis of jejunal villus height/crypt depth ratio (V/C). (**G**) Serum IL-6 content. (**H**) Serum IL-10 content. (**I**) Serum IgG content. CK: control group; DON: DON model group; L-FME: low-dose FME intervention group; M-FME: medium-dose FME intervention group; H-FME: high-dose FME intervention group. Different lowercase letters indicate significant differences among groups (*p* < 0.05).

After 1 week of acclimatization, sixty mice were randomly divided into 5 groups, with 12 mice per group (*n* = 12 per group), as follows: control group (CK): received normal saline; DON model group (DON): 3 mg/kg body weight (BW) DON; lLow-dose FME + DON group (L-FME): 25 mg/kg BW FME + 3 mg/kg BW DON; medium-dose FME + DON group (M-FME): 50 mg/kg BW FME + 3 mg/kg BW DON; high-dose FME + DON group (H-FME): 100 mg/kg BW FME + 3 mg/kg BW DON. The doses of FME were selected based on previous studies on the protective effects of FME [17]. FME (suspended in sterile saline) and DON (dissolved in sterile saline) were administered daily by oral gavage for 4 weeks, with FME given 8 h prior to DON treatment [24]. After 4 consecutive weeks of intervention, mice were anesthetized with sodium pentobarbital (50 mg/kg, administered intraperitoneally) and then sacrificed by cervical dislocation. (Figure 1A). Blood samples were collected from the retro-orbital venous plexus, and serum was separated. Liver and jejunum tissues were rapidly dissected and rinsed with sterile saline. Partial jejunum tissues were fixed with 4% fresh paraformaldehyde for histomorphological observation, and subsamples of intestinal tissues were stored at −80 °C for subsequent analyses.

### 2.3. Measurement of Body Weight

Body weight was recorded daily at a fixed time using an electronic balance. Each mouse was weighed three times consecutively, and the average value was calculated to minimize measurement error and improve accuracy and repeatability.

### 2.4. Histopathological Analysis

Fresh liver and jejunum were fixed, trimmed, dehydrated, paraffin-embedded, and sectioned using 4% paraformaldehyde. Tissue blocks were processed through graded ethanol series and xylene for optimal preservation of cellular morphology. Sections were H&E-stained and then examined under light microscopy to capture representative histological images.

### 2.5. ELISA Analysis of Inflammatory Cytokines

Serum levels of interleukin-6 (IL-6, Cat. No. JLW20268), interleukin-10 (IL-10, Cat. No. JLW20242), and immunoglobulin G (IgG, Cat. No. JL12948) were measured using commercial ELISA kits (Jianglai Biotechnology, Shanghai, China) strictly following the manufacturer’s protocols. All samples were assayed in duplicate, and the mean values were used for statistical analysis. The absorbance was read at 450 nm using a microplate reader, and the cytokine concentrations were calculated against standard curves generated with serial dilutions of the provided standards.

### 2.6. Detection of Constituents in FME by UPLC-QTOF-MS

The Fructus mume extract (FME) was dissolved in methanol, ultrasonicated for 60 min, and then centrifuged. The supernatant was collected, diluted 10,000-fold, and filtered through a 0.22 μm microporous membrane prior to analysis. Qualitative analysis of the chemical components in FME was performed using ultra-performance liquid chromatography coupled with quadrupole time-of-flight mass spectrometry (UPLC-Q-TOF-MS). Chromatographic separation was achieved on an ACQUITY UPLC HSS T3 column (2.1 mm × 100 mm, 1.8 μm) with gradient elution using 0.1% formic acid in water (phase A) and acetonitrile (phase B) as the mobile phase at a flow rate of 0.3 mL/min. The column temperature was maintained at 40 °C, and the injection volume was 2 μL. Mass spectrometry was conducted in electrospray ionization (ESI) negative-ion mode over a mass range of *m*/*z* 50–1500, with a capillary voltage of 2.5 kV, an ion source temperature of 120 °C, a desolvation gas temperature of 350 °C, and a desolvation gas flow rate of 600 L/h. Identification of the chemical constituents was accomplished by matching the retention times, accurate molecular masses, and MS/MS fragmentation patterns against the ChemSpider database.

### 2.7. Network Pharmacology Analysis

The components identified by mass spectrometry analysis. Candidate components were screened with oral bioavailability (OB) ≥ 30% and drug-likeness (DL) ≥ 0.18. These criteria are widely used in network pharmacology to ensure the selection of components with favorable pharmacokinetic profiles. Structural information was obtained from the PubChem (https://pubchem.ncbi.nlm.nih.gov/, accessed on 20 December 2025) database. Potential targets were predicted using Swiss Target Prediction (https://swisstargetprediction.ch/, accessed on 20 December 2025) (Probability ≥ 0.1), the Similarity Ensemble Approach (SEA, *p* < 0.05), PharmMapper (https://www.lilab-ecust.cn/pharmmapper/index.html, accessed on 20 December 2025), and TargetNet (http://targetnet.scbdd.com/, accessed on 20 December 2025). The intersection of predicted targets was defined as a set of high-confidence candidate targets for *Fructus mume*.

Disease targets associated with liver and intestinal injury were retrieved from the DrugBank, GeneCards, OMIM, and PharmGKB databases. Duplicate entries were removed to obtain a unified target set. Overlapping targets between drug candidates and diseases were identified with Venny 2.1.0 and defined as core therapeutic targets.

To identify key active components, we constructed and analyzed a component-target network using Cytoscape (v3.9.1). A protein–protein interaction (PPI) network was then generated from the STRING database (https://string-db.org/, accessed on 21 December 2025) (confidence score > 0.4, disconnected nodes removed) and visualized in Cytoscape. Core hub proteins were screened using the CytoHubba plugin.

GO functional enrichment analysis and KEGG enrichment analysis were conducted using the DAVID online tool (https://davidbioinformatics.nih.gov/, accessed on 21 December 2025). Results were visualized using a bioinformatics platform to generate GO and KEGG plots.

### 2.8. Determination of Hepatic Oxidative Stress Markers

Commercial kits purchased from Grace Biotechnology (Suzhou, China) were applied to measure hepatic levels of five indicators: MDA (Cat. No. G0109W), GSH (G0206W), T-AOC (G0115W), CAT (G0105W), and SOD (G0101W). The corresponding absorbance values were recorded at 532, 412, 590, 510, and 450 nm, respectively. A Pierce BCA Protein Assay Kit (Abbkine, Wuhan, China) was used to quantify total protein concentration.

### 2.9. Quantitative Real-Time Polymerase Chain Reaction (qRT-PCR)

The total RNA of the liver and the jejunum was extracted using the AG RNAex Pro Kit (Hunan Accurate Biological Engineering Co., Ltd., Changsha, China). OD_260_/OD_280_ was used as a criterion to assess RNA purity. Reverse transcription of total RNA to cDNA employed the Evo M-MLV Kit (Hunan Accurate Biological Engineering Co., Ltd., Changsha, China). Triplicate qRT-PCR for each sample was carried out on a real-time fluorescent quantitative analyzer. The amplification protocol consisted of reverse transcription (42 °C, 5 min), pre-denaturation (95 °C, 10 min), and 40 cycles of 95 °C for 10 s and 60 °C for 35 s. Relative mRNA levels were determined via the 2^−ΔΔCt^ method, β-actin was employed as the reference gene for normalization. Primers were synthesized by Sangon Biotech (Shanghai, China). Their sequences are listed in Table 1.

### 2.10. TUNEL Assay for Jejunal Apoptosis

After treatment, mice were euthanized, and the jejunum was harvested. The tissues were placed in paraformaldehyde for 24 h, followed by repeated rinsing with distilled water, dehydration, paraffin embedding, and sectioning. TUNEL staining was carried out, and fluorescence microscopy was used to acquire images. The number of TUNEL-positive cells was counted in randomly selected fields per section to evaluate apoptosis.

### 2.11. Immunohistochemistry (IHC) Assay

Tissue specimens were kept in 4% formaldehyde (pH 7.4) at 4 °C for 48 h, followed by dehydration, paraffin embedding, and sectioning at 4 μm thickness. Antigen retrieval was achieved using the EDTA retrieval buffer (Abcam, Cambridge, UK). The sections were incubated overnight (4 °C) with primary antibodies (Servicebio, Wuhan, China) against Claudin 1 (1:1000), Occludin (1:6000), and ZO-1 (1:1300). Subsequent procedures were conducted as specified by the MaxVision HRP IHC kit (Fuzhou Maixin Biotechnology Development Co., Ltd., Fuzho, China). DAB staining, hematoxylin counterstaining, dehydration, and mounting were then performed. Positive expression in the jejunum was observed under a light microscope.

### 2.12. Sequencing the Metagenome of Fecal Microbiota

After being thawed at room temperature, the frozen cecal fecal samples from five mice per group (*n* = 5) were used to extract total microbial DNA. This extraction was performed with the QIAGEN Fecal DNA Extraction Kit (QIAGEN Biotechnology (Shenzhen) Co., Ltd., Shenzhen, China), following the manufacturer’s protocol, and the extracted material was dissolved in sterile ultrapure water and stored at −20 °C. DNA purity was examined using a Nanodrop spectrophotometer (Thermo Fisher Scientific Inc., Waltham, MA, USA). For microbiome analysis, the V3–V4 region of the 16S rRNA gene was sequenced on an Illumina NovaSeq platform (Illumina, Inc., San Diego, CA, USA), generating raw reads per sample. Qualified DNA underwent library preparation using the Rapid Barcoding Kit V14 (SQK RBK114.24/114.96) and was subsequently sequenced on an Illumina NovaSeq platform with PE150 paired-end reads.

Raw reads were filtered using fastp (v0.23.4) to remove low-quality reads, adapters, and contaminants. Host DNA was removed by mapping clean reads to the mouse reference genome using Bowtie2 (v2.5.1). Taxonomic annotation was performed using Kraken2 (v2.1.2) and Bracken (v2.8) to determine microbial composition and relative abundance at the phylum, class, order, family, genus, and species levels. Rarefaction curves, alpha diversity (Shannon, Chao1), and beta diversity (PCoA) were analyzed and plotted in R (v4.3.2).

### 2.13. Liver Metabolomics Analysis

Frozen liver samples from five mice per group (*n* = 5) were thawed slowly on ice. Exactly 30 mg of liver tissue was placed in a sterile grinding tube, mixed with 600 μL of prechilled methanol–acetonitrile–water (2:2:1, *v*/*v*/*v*), and spiked with 10 μL of internal standard (L-2-chlorophenylalanine, 1 μg/mL) to correct extraction efficiency and instrumental drift. Samples were homogenized at 4 °C and 60 Hz for 3 min, incubated at 4 °C for 30 min, and centrifuged at 12,000 r/min for 15 min at 4 °C. The supernatant was collected, and the pellet was re-extracted with 300 μL of the same solvent. Supernatants were combined, vortexed for 30 s, and lyophilized. The dried residue was reconstituted in 100 μL of a methanol–water mixture (1:1, *v*/*v*), then vortexed for 1 min and spun at 12,000 r/min for 10 min at 4 °C. The resulting supernatant was filtered through a 0.22 μm organic filter and stored at 4 °C until analysis. All operations were performed at low temperature to prevent metabolite oxidation and degradation. Reagents were of chromatographic grade, and utensils were autoclaved and dried to avoid contamination.

Metabolite quantification was performed using a UHPLC-MS/MS system. Chromatographic system: an ACQUITY UPLC HSS T3 (100 × 2.1 mm, 1.8 μm) column at 40 °C; 5 μL injection, 0.3 mL/min. Mobile phases: 0.1% formic acid in water (A) and acetonitrile (B). Gradient elution: 5%B initially (0–1min), linear to 95%B (1–9 min), 95%B (9–10 min), linear to 5%B (10–11 min), re-equilibration at 5%B (11–14 min).

Mass spectrometry was performed via electrospray ionization (ESI) in the positive- and negative-ion modes. The ion source temperature and the desolvation temperature were 150 °C and 500 °C, respectively. The desolvation gas flow rate and the cone gas flow were 800 L/h and 50 L/h, respectively. Detection was performed in multiple reaction monitoring (MRM) mode. The mass range was *m/z* 50–1000, the collision gas was argon, and the collision energy was optimized for each metabolite. Quality control (QC) samples (pooled from all samples) were injected every 10 injections to monitor system stability.

Raw data processing (peak detection, alignment, noise reduction, integration, and internal standard correction) was performed with Progenesis QI software (v3.0; Waters Corporation, Milford, MA, USA). All peaks with a signal-to-noise ratio (S/N) < 3 were deleted. The peak area matrix was exported, normalized, and subjected to further bioinformatics analysis.

PCA and OPLS-DA were conducted with SIMCA 14.1, and R. Metabolites showing VIP > 1 and *p* < 0.05 were considered differential. Annotation of metabolite names, categories, and functions was carried out using HMDB (https://hmdb.ca/, accessed on 13 January 2026).and Metlin (https://ngdc.cncb.ac.cn, accessed on 13 January 2026). For metabolomics analysis, QC samples were prepared by pooling equal volumes of all samples and injecting every injection to monitor system stability. Data preprocessing, including peak alignment, baseline correction, and total ion current normalization, was performed using Progenesis QI.

### 2.14. Statistical Analysis

Data are presented as mean ± SEM. GraphPad Prism 10.1.2 or SPSS (v29.0; IBM Corp., Armonk, NY, USA) 2022 was used to conduct statistical tests and generate plots. Prior to analysis, the normality of data distribution was assessed using the Shapiro–Wilk test and the homogeneity of variances was verified using Levene’s test. In addition, one-way ANOVA with Tukey’s post hoc test was used. *p* < 0.05 was considered statistically significant.

## 3. Results

### 3.1. Effects of FME on Growth Performance, Histopathology, and Inflammatory-Immune Parameters in DON-Exposed Mice

**Figure 2:**
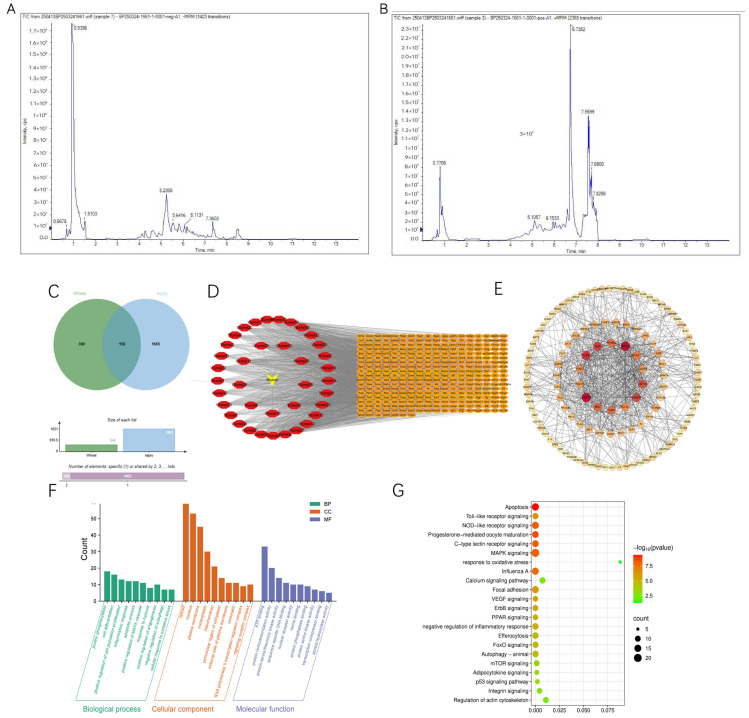
UPLC-Q-TOF-MS analysis of FME and network pharmacology study against DON-induced injury. (**A**) Total ion chromatogram (TIC) of FME in positive-ion mode. (**B**) TIC of FME in negative-ion mode. The abscissa indicates retention time (min), and the ordinate indicates signal intensity (cps). Scan ranges were set at *m*/*z* 100–1500 for TOF MS and *m*/*z* 50–1500 for TOF MS/MS. (**C**) Venn diagram of FME active component targets and DON-induced injury targets. (**D**) Active component-core target regulatory network of FME. (**E**) Protein–protein interaction (PPI) network of core targets. (**F**) GO functional enrichment analysis of core targets (BP: biological process, CC: cellular component, MF: molecular function, ES: enrichment score). (**G**) Bubble plot of key biological-process enrichment for core targets. Dot color represents enrichment significance (−log_10_(*p*-value)), and dot size represents the number of enriched genes (count).

Body weight was normalized to the initial body weight of each mouse and expressed as a percentage (Figure 2B). At baseline, no significant difference in body weight was observed among the groups (*p* > 0.05). At day 27, the body weight percentage in the DON group was significantly lower than that in the CK group (106.43 ± 1.13% vs. 115.56 ± 0.03%, *p* < 0.05), indicating that DON exposure suppressed body weight gain. Compared with the DON group, M-FME and H-FME treatment significantly increased the body weight percentage to 111.89 ± 0.62% and 115.76 ± 1.26%, respectively (*p* < 0.05), whereas the L-FME group showed no significant difference from the DON group (107.43 ± 1.31%, *p* > 0.05). Notably, the H-FME group was comparable to the CK group at day 27 (*p* > 0.05). These results suggest that FME, especially at medium and high doses, attenuated DON-induced suppression of body weight gain.

Representative H&E-stained liver and jejunal sections are shown in Figure 1C. In the CK group, the hepatic lobule structure was intact, hepatocytes were regularly arranged, and no obvious inflammatory infiltration or necrosis was observed. In contrast, DON exposure induced evident hepatic histopathological alterations, including disordered hepatic lobule structure, hepatocyte swelling and vacuolar degeneration, and inflammatory cell infiltration. Compared with the DON group, FME-treated groups showed alleviated hepatic histopathological injury, with the most obvious improvement observed in the H-FME group.

In the jejunum, the CK group showed intact and well-arranged villi with normal crypt morphology. The DON group exhibited obvious jejunal mucosal injury, characterized by villus shortening, villus fracture or lodging, increased crypt depth, and impaired mucosal integrity. FME treatment alleviated these DON-induced jejunal histopathological changes, particularly in the H-FME group.

Quantitative analysis of jejunal villus height, crypt depth, and the villus height/crypt depth ratio (V/C ratio) is shown in Figure 1D–F. Compared with the CK group, DON exposure significantly reduced villus height and the V/C ratio, while significantly increasing crypt depth (*p* < 0.05). Compared with the DON group, FME treatment increased villus height and the V/C ratio and reduced crypt depth (*p* < 0.05), with the most pronounced effect observed in the H-FME group. These results suggest that FME alleviated DON-induced jejunal structural damage.

Serum IL-6, IL-10, and IgG levels are shown in Figure 1G–I. Compared with the CK group, DON exposure significantly increased serum IL-6 levels (*p* < 0.01), whereas FME treatment reduced IL-6 levels compared with the DON group (*p* < 0.05). Serum IL-10 levels were significantly lower in the DON group than in the CK group (*p* < 0.01), while FME treatment increased IL-10 levels compared with the DON group (*p* < 0.05). In addition, DON exposure significantly reduced serum IgG levels compared with the CK group (*p* < 0.01), and FME treatment increased IgG levels compared with the DON group (*p* < 0.05). These findings indicate that FME attenuated DON-induced alterations in inflammatory cytokines and humoral immune-related IgG levels.

### 3.2. Potential Targets and Functional Enrichment Analysis of FME Against DON-Induced Injury

As shown in Figure 2A,B, all peaks in the TIC of FME under positive- and negative-ion modes were eluted within 14 min, indicating effective detection of most constituents. By matching against the SCIEX OS database with auxiliary identification, 38 compounds were identified (Table 2).

The active components of FME identified by UPLC yielded 546 associated targets, and network pharmacology analysis retrieved 1821 DON-induced intestinal/liver injury-related targets. A total of 156 overlapping targets were obtained and considered candidate targets for subsequent analysis (Figure 2C).

Based on the 156 overlapping targets, the active components–core targets regulatory network of FME was constructed (Figure 2D). The left nodes represent the active components of FME, and the right nodes represent the core targets; the connecting lines indicate the regulatory relationships between components and targets, visually demonstrating the multi-component and multi-target synergistic effects of FME.

Furthermore, the overlapping targets were submitted to the STRING database for PPI network construction (Figure 2E). The red nodes represent the core hub targets with the highest degree values, such as IL-1β, IL-6, and caspase3, suggesting that these targets are key molecules for the anti-inflammatory, antioxidant, and tissue repair effects of FME.

GO functional enrichment analysis of the 156 core overlapping targets was performed (Figure 2F). The biological processes targeted were significantly enriched in apoptotic process, inflammatory response, and cellular response to oxidative stress, indicating that FME alleviates DON-induced tissue injury by regulating biological processes related to apoptotic, inflammation, and oxidative stress. The cell components targeted were mainly enriched in the cell membrane, cytoplasm, nucleus, and organelle membrane, which is highly consistent with the subcellular localization of DON-induced injury.

Molecular function targets were significantly enriched in nuclear receptor activity, ATP binding, heme binding, and steroid hydroxylase activity, providing a molecular basis for FME in regulating cell signal transduction and inflammatory pathway activation. Further visualization of biological-process enrichment was performed (Figure 2G). The core targets of FME were most closely linked to enhancing protein kinase activity and protein phosphorylation, suppressing inflammatory responses, counteracting oxidative stress, and inhibiting apoptosis.

### 3.3. Effects of FME on Hepatic Oxidative Stress and Inflammation-Related Parameters in DON-Exposed Mice

**Figure 3:**
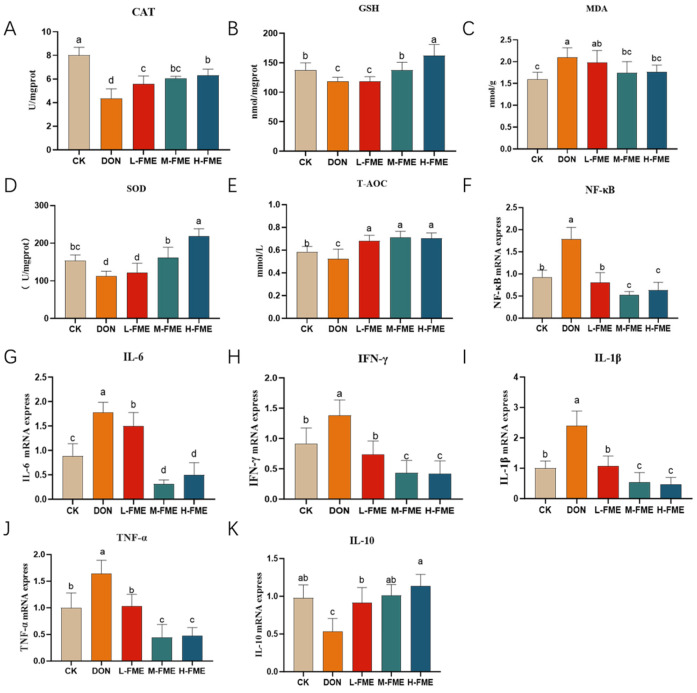
Effects of *Fructus mume* extract (FME) on hepatic oxidative stress and inflammation-related indicators in deoxynivalenol (DON)-exposed mice. Note: (**A**) catalase (CAT) activity; (**B**) glutathione (GSH) content; (**C**) malondialdehyde (MDA) content; (**D**) superoxide dismutase (SOD) activity; (**E**) total antioxidant capacity (T-AOC); (**F**) relative mRNA expression of NF-κB; (**G**) relative mRNA expression of IL-6; (**H**) relative mRNA expression of IFN-γ; (**I**) relative mRNA expression of IL-1β; (**J**) relative mRNA expression of TNF-α; (**K**) relative mRNA expression of IL-10. CK: control group; DON: DON model group; L-FME: low-dose FME group; M-FME: medium-dose FME group; H-FME: high-dose FME group. Different lowercase letters indicate significant differences among groups (*p* < 0.05). Data are presented as mean ± SEM, *n* = 10.

Hepatic oxidative stress markers are shown in Figure 3A–E. Compared with the CK group, DON exposure significantly decreased hepatic CAT, GSH, SOD, and T-AOC levels, while significantly increasing MDA levels (*p* < 0.01). Compared with the DON group, FME treatment increased hepatic CAT, GSH, SOD, and T-AOC levels and reduced MDA levels (*p* < 0.05), with the most pronounced effects observed in the H-FME group. In the H-FME group, CAT, SOD, and T-AOC levels were not significantly different from those in the CK group (*p* > 0.05), while GSH levels were higher than those in the CK group (*p* < 0.05). These results suggest that FME alleviated DON-induced hepatic oxidative stress.

Hepatic mRNA expression levels of NF-κB and inflammation-related factors are shown in Figure 3F–K. Compared with the CK group, DON exposure significantly increased the mRNA expression levels of NF-κB, IL-6, IFN-γ, IL-1β, and TNF-α, while decreasing IL-10 expression (*p* < 0.05). Compared with the DON group, FME treatment reduced the expression of NF-κB and pro-inflammatory cytokines and increased IL-10 expression (*p* < 0.05), particularly in the H-FME group. These results suggest that FME may alleviate DON-induced hepatic inflammatory responses at the transcriptional level.

### 3.4. Effects of FME on Jejunal Mucosal Barrier Function and Apoptosis-Related Gene Expression in DON-Exposed Mice

**Figure 4:**
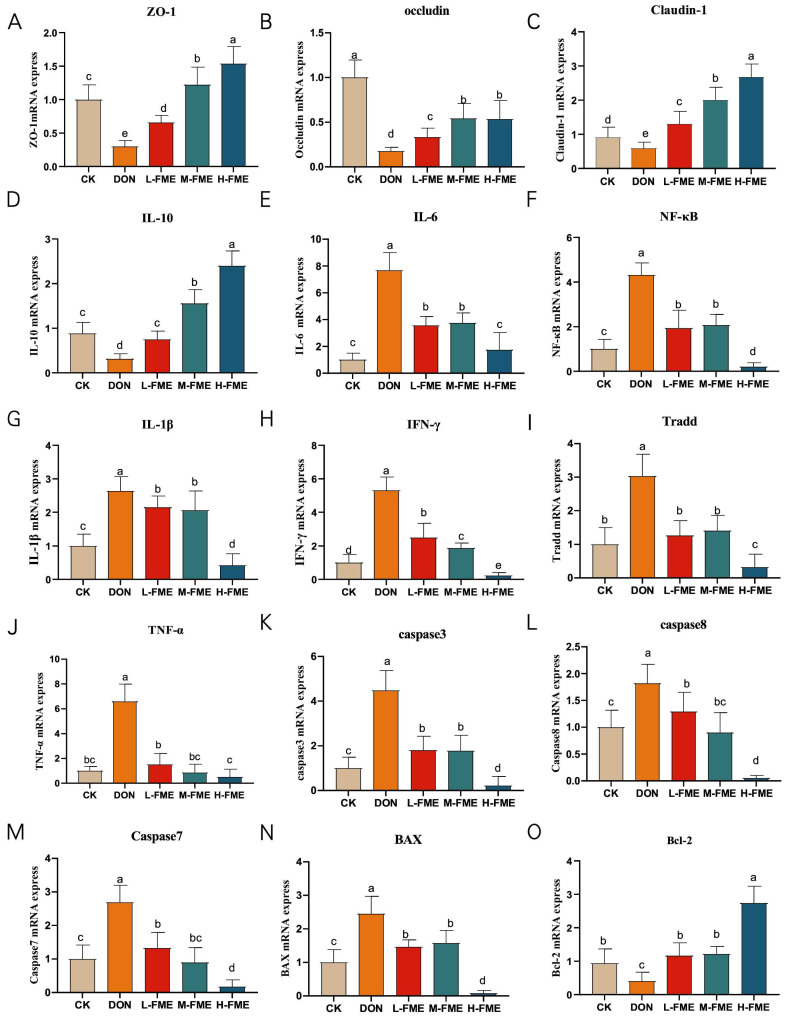
Effects of *Fructus mume* extract (FME) on mRNA expression of jejunal tight junction, inflammation, and apoptosis-related genes in deoxynivalenol (DON)-exposed mice. (**A**) Relative mRNA expression of *ZO-1*; (**B**) relative mRNA expression of *Occludin*; (**C**) relative mRNA expression of *Claudin-1*; (**D**) relative mRNA expression of *IL-10*; (**E**) relative mRNA expression of *IL-6*; (**F**) relative mRNA expression of *NF-κB*; (**G**) relative mRNA expression of *IL-1β*; (**H**) relative mRNA expression of *IFN-γ*; (**I**) relative mRNA expression of Tradd; (**J**) relative mRNA expression of *TNF-α*; (**K**) relative mRNA expression of *Caspase-3*; (**L**) relative mRNA expression of *Caspase-8*; (**M**) relative mRNA expression of *Caspase-7*; (**N**) relative mRNA expression of *BAX*; (**O**) relative mRNA expression of *Bcl-2*. CK: control group; DON: DON model group; L-FME: low-dose FME group; M-FME: medium-dose FME group; H-FME: high-dose FME group. Different lowercase letters indicate significant differences among groups (*p* < 0.05). Data are presented as mean ± SEM, *n* = 10.

Jejunal mRNA expression levels of tight junction-, inflammation-, and apoptosis-related genes are shown in Figure 4. Compared with the CK group, DON exposure significantly decreased the mRNA expression levels of *ZO-1*, *Occludin*, and *Claudin-1* in the jejunum (*p* < 0.01; Figure 4A–C). FME treatment increased the expression of these tight junction-related genes compared with the DON group (*p* < 0.05). In particular, the expression levels of *ZO-1*, *Occludin*, and *Claudin-1* in the H-FME group were not significantly different from those in the CK group (*p* > 0.05), suggesting that FME attenuated DON-induced downregulation of tight junction-related genes.

Inflammation-related gene expression is shown in Figure 4D–I. Compared with the CK group, DON significantly decreased jejunal *IL-10* mRNA expression and increased the mRNA expression levels of *IL-6*, *NF-κB*, *IL-1β*, *IFN-γ*, and *TNF-α* (*p* < 0.01). FME treatment increased *IL-10* expression and reduced the expression of DON-induced pro-inflammatory factors and *NF-κB* compared with the DON group (*p* < 0.05). Among the treatment groups, H-FME showed the most pronounced effect, with significantly lower *IL-6*, *NF-κB*, *IL-1β*, *IFN-*γ, and *TNF-α* expression than the DON group (*p* < 0.01).

The mRNA expression levels of apoptosis-related genes are shown in Figure 4J–O. DON exposure significantly increased the jejunal mRNA expression levels of *Tradd*, *Caspase-3*, *Caspase-7*, *Caspase-8*, and *BAX*, while decreasing *Bcl-2* expression, compared with the CK group (*p* < 0.05 or *p* < 0.01). Compared with the DON group, FME treatment reduced the expression of pro-apoptotic genes and increased *Bcl-2* expression (*p* < 0.05), particularly in the H-FME group. These results suggest that FME may modulate DON-induced alterations in the expression of genes related to tight junctions, inflammation, and apoptosis in the jejunum.

### 3.5. Effects of FME on Jejunal Apoptosis and Tight Junction Protein Expression in DON-Exposed Mice

**Figure 5:**
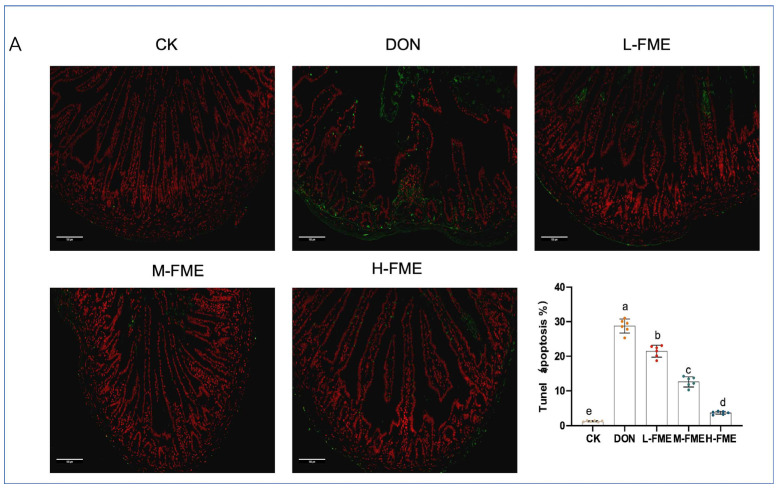

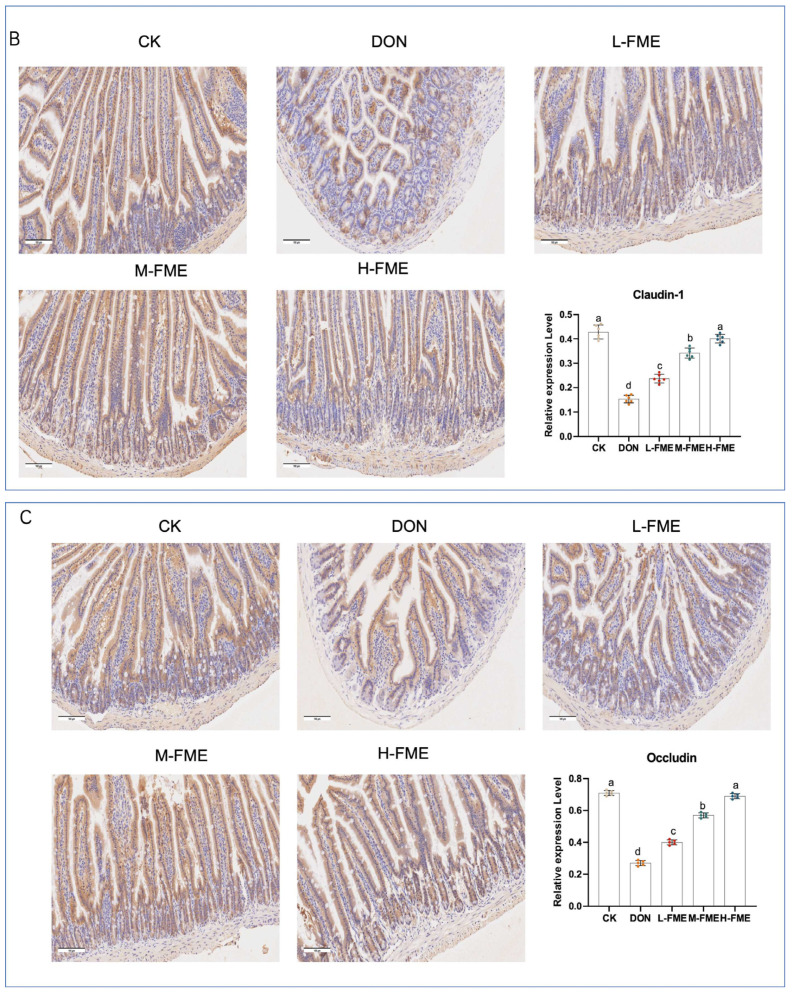

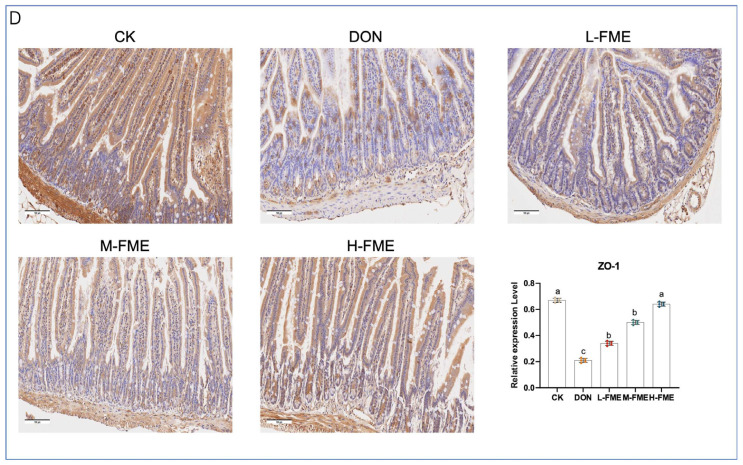
Effects of *Fructus mume* extract (FME) on jejunal cell apoptosis and tight junction protein expression in deoxynivalenol (DON)-exposed mice. (**A**) TUNEL staining of jejunal tissue (green fluorescence indicates apoptotic cells) and quantification of apoptosis rate; (**B**) immunohistochemical staining and relative expression of Claudin-1; (**C**) immunohistochemical staining and relative expression of Occludin; (**D**) immunohistochemical staining and relative expression of ZO-1. CK: control group; DON: DON model group; L-FME: low-dose FME group; M-FME: medium-dose FME group; H-FME: high-dose FME group. Different lowercase letters indicate significant differences among groups (*p* < 0.05). Data are presented as mean ± SEM, *n* = 6. Scale bar: 100 μm.

TUNEL staining showed changes in jejunal apoptosis among the different groups (Figure 5A). In the CK group, only a few TUNEL-positive cells were observed in the jejunal epithelium. Compared with the CK group, DON exposure markedly increased the number of TUNEL-positive cells and significantly increased the apoptotic rate in the jejunum (*p* < 0.01). FME treatment reduced the number of TUNEL-positive cells and decreased the apoptotic rate compared with the DON group (*p* < 0.05). In particular, the apoptotic rate in the H-FME group was not significantly different from that in the CK group (*p* > 0.05). These results suggest that FME attenuated DON-induced jejunal apoptosis.

The immunohistochemical staining results of Claudin-1, Occludin, and ZO-1 are shown in Figure 5B–D. In the CK group, positive staining for these tight junction proteins was clearly observed in the jejunal mucosal epithelium. Compared with the CK group, DON exposure significantly reduced the expression levels of Claudin-1, Occludin, and ZO-1 (*p* < 0.01), accompanied by weaker staining intensity. FME treatment increased the expression of these tight junction proteins compared with the DON group (*p* < 0.05), with the most obvious improvement observed in the H-FME group. The expression levels of Claudin-1, Occludin, and ZO-1 in the H-FME group were not significantly different from those in the CK group (*p* > 0.05). These results suggest that FME may alleviate DON-induced decreases in jejunal tight junction protein expression, in line with the qRT-PCR findings for tight junction-related genes.

### 3.6. Effects of FME on Gut Microbiota Diversity and Community Structure in DON-Exposed Mice

**Figure 6:**
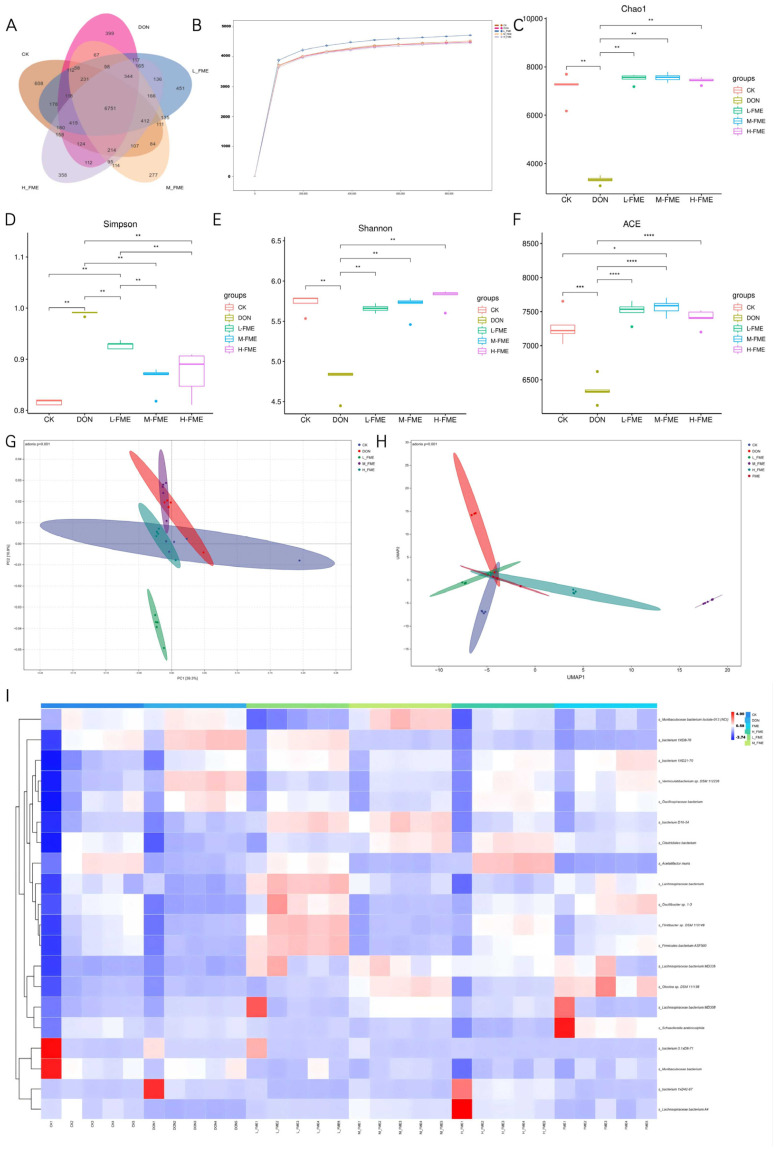
Effects of *Fructus mume* extract (FME) on gut microbiota diversity and community structure in deoxynivalenol (DON)-exposed mice. (**A**) Venn diagram of OTUs in each group, different colors represent different groups; (**B**) rarefaction curves, different colors represent different groups; (**C**) Chao1 index; (**D**) Simpson index; (**E**) Shannon index; (**F**) ACE index; (**G**) PCoA analysis based on Bray–Curtis distance; (**H**) UMAP analysis; (**I**) heatmap of gut microbiota composition at the genus level. CK: control group; DON: DON model group; L-FME: low-dose FME group; M-FME: medium-dose FME group; H-FME: high-dose FME group. * *p* < 0.05, ** *p* < 0.01, *** *p* < 0.001, **** *p* < 0.0001. Data are presented as mean ± SEM, *n* = 6.

The diversity and composition of the cecal microbiota are shown in Figure 6. The Venn diagram shows that the numbers of unique OTUs in the CK, DON, L-FME, M-FME, and H-FME groups were 608, 399, 451, 277, and 358, respectively, with 6751 OTUs shared among groups (Figure 6A). The rarefaction curves tended to reach a plateau, indicating that the sequencing depth was sufficient for subsequent microbiota analysis (Figure 6B).

Alpha diversity indices, including Chao1, Simpson, Shannon, and ACE, are shown in Figure 6C–F. Compared with the CK group, DON exposure significantly decreased the Chao1, ACE, and Shannon indices and increased the Simpson index (*p* < 0.01), indicating reduced microbial richness and diversity. Compared with the DON group, FME treatment increased the Chao1, ACE, and Shannon indices and decreased the Simpson index (*p* < 0.05), with the most pronounced changes observed in the H-FME group.

Beta diversity analysis is shown in Figure 6G,H. PCoA based on the Bray–Curtis distance showed a clear separation between the CK and DON groups, suggesting that DON exposure altered the overall microbial community structure. FME-treated groups shifted toward the CK group, particularly the H-FME group. Similar clustering patterns were also observed in the UMAP analysis, indicating that FME treatment was associated with partial recovery of DON-induced microbiota disturbance.

The genus-level heatmap showed differences in microbial composition among groups (Figure 6I). DON exposure altered the relative abundance of several taxa, including *Schroederella arabinoxylan*, *Verminatibacterium* sp. *DSM 112226*, *Lactobacillus acidophilus*, and *Lachnospiraceae bacterium*. Compared with the DON group, FME treatment modified the abundance patterns of these taxa, and the H-FME group showed a microbial profile closer to that of the CK group.

### 3.7. Effects of FME on Gut Microbiota Composition at Phylum, Family, and Genus Levels

**Figure 7:**
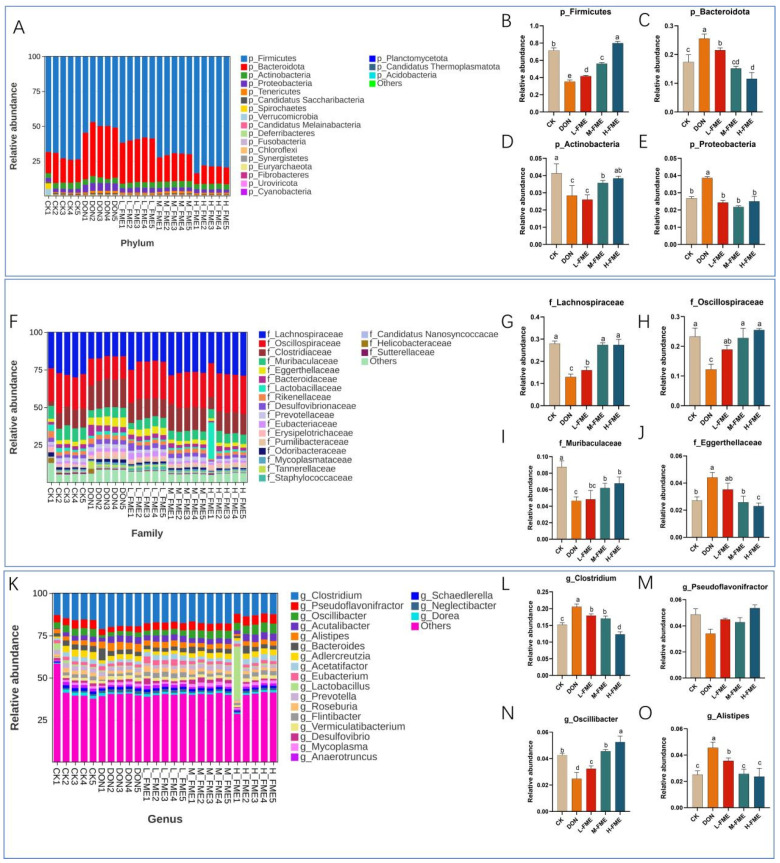
Effects of *Fructus mume* extract (FME) on gut microbiota composition at phylum, family, and genus levels in deoxynivalenol (DON)-exposed mice. (**A**) Stacked bar plot of phylum-level composition; (**B**) abundance of Firmicutes; (**C**) abundance of Bacteroidota; (**D**) abundance of Actinobacteriota; (**E**) abundance of Proteobacteria; (**F**) stacked bar plot of family-level composition; (**G**) abundance of Lachnospiraceae; (**H**) abundance of Oscillospiraceae; (**I**) abundance of Muribaculaceae; (**J**) abundance of Eggerthellaceae; (**K**) stacked bar plot of genus-level composition; (**L**) abundance of Clostridium; (**M**) abundance of Pseudoflavonifractor; (**N**) abundance of Oscillibacter; (**O**) abundance of Alistipes. CK: control group; DON: DON model group; L-FME: low-dose FME group; M-FME: medium-dose FME group; H-FME: high-dose FME group. Different lowercase letters indicate significant differences among groups (*p* < 0.05). Data are presented as mean ± SEM, *n* = 6.

The relative abundance of cecal microbiota at the phylum, family, and genus levels is shown in Figure 7. At the phylum level, Firmicutes, Bacteroidota, Actinobacteriota, and Proteobacteria were the dominant bacterial phyla, together accounting for more than 95% of the total microbiota. Compared with the CK group, DON exposure significantly decreased the relative abundance of Firmicutes and Actinobacteriota and increased the relative abundance of Bacteroidota and Proteobacteria (*p* < 0.01), resulting in a reduced Firmicutes/Bacteroidota ratio. Compared with the DON group, FME treatment partially reversed these changes, with the most obvious effect observed in the H-FME group.

At the family level, DON exposure decreased the relative abundance of Lachnospiraceae, Oscillospiraceae, and Muribaculaceae, while increasing the relative abundance of Eggerthellaceae (*p* < 0.05). FME treatment modified these DON-induced changes in family-level microbiota composition, particularly in the M-FME and H-FME groups.

At the genus level, DON exposure significantly reduced the relative abundance of *Clostridium*, *Oscillibacter*, *Pseudoflavonifractor*, and *Alistipes* (*p* < 0.05). Compared with the DON group, FME treatment increased the relative abundance of these genera, with the H-FME group showing a microbial composition closer to that of the CK group. These results suggest that FME may partially restore DON-induced alterations in cecal microbiota composition at multiple taxonomic levels.

### 3.8. Effects of FME on Hepatic Metabolomic Profiles in DON-Exposed Mice

**Figure 8:**
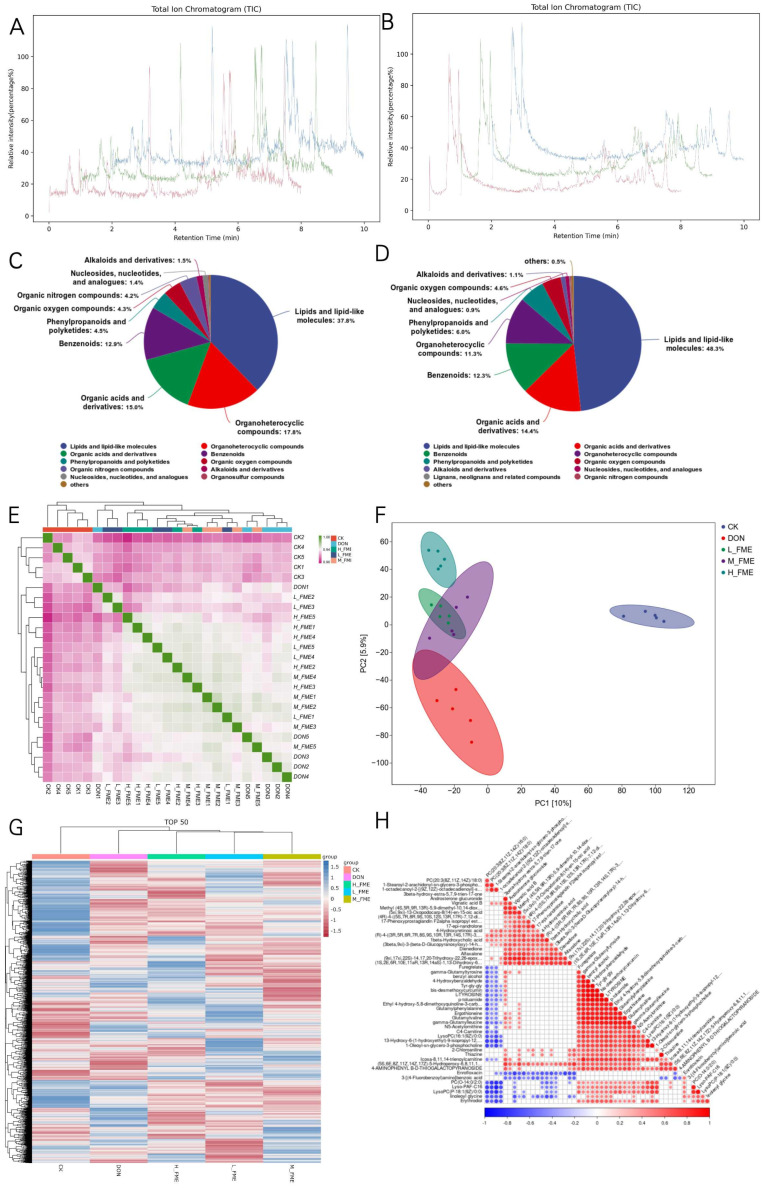
FME on hepatic metabolomic profiles in DON-exposed mice. (**A**) TICs of liver metabolites; (**B**) classification of liver metabolites; (**C**,**D**) proportions of metabolite categories; (**E**) clustering heatmap of liver metabolites; (**F**) PCA score plot; (**G**) heatmap of TOP50 differential metabolites; (**H**) correlation heatmap of differential metabolites. CK: control group; DON: DON model group; L-FME: low-dose FME group; M-FME: medium-dose FME group; H-FME: high-dose FME group.

The quality control results of the non-targeted LC-MS metabolomics analysis are shown in Figure 8A,B. The total ion chromatograms (TICs) show stable signal intensity and good peak separation across samples, indicating acceptable data quality for subsequent metabolomic analysis. Metabolite classification revealed that DON exposure dramatically increased levels of lipids and lipid-like molecules and decreased levels of organic acids and derivatives. FME intervention restored the metabolic profile toward the CK group (Figure 8C,D).

Hierarchical clustering heatmap (Figure 8E) showed distinct separation between the CK and DON groups, while FME-treated groups clustered closer to CK. The PCA score plot (Figure 8F) shows clear separation among groups, with FME shifting metabolic profiles toward control in a dose-dependent manner.

The TOP50 differential metabolite heatmap (Figure 8G) shows that DON upregulated pro-inflammatory and oxidative stress-related metabolites and downregulated protective metabolites. FME reversed these changes. Correlation analysis (Figure 8H) showed that FME restructured the metabolic network by weakening pro-damage correlations and strengthening beneficial interactions.

### 3.9. Effects of FME on Key Hepatic Differential Metabolites in DON-Exposed Mice

**Figure 9:**
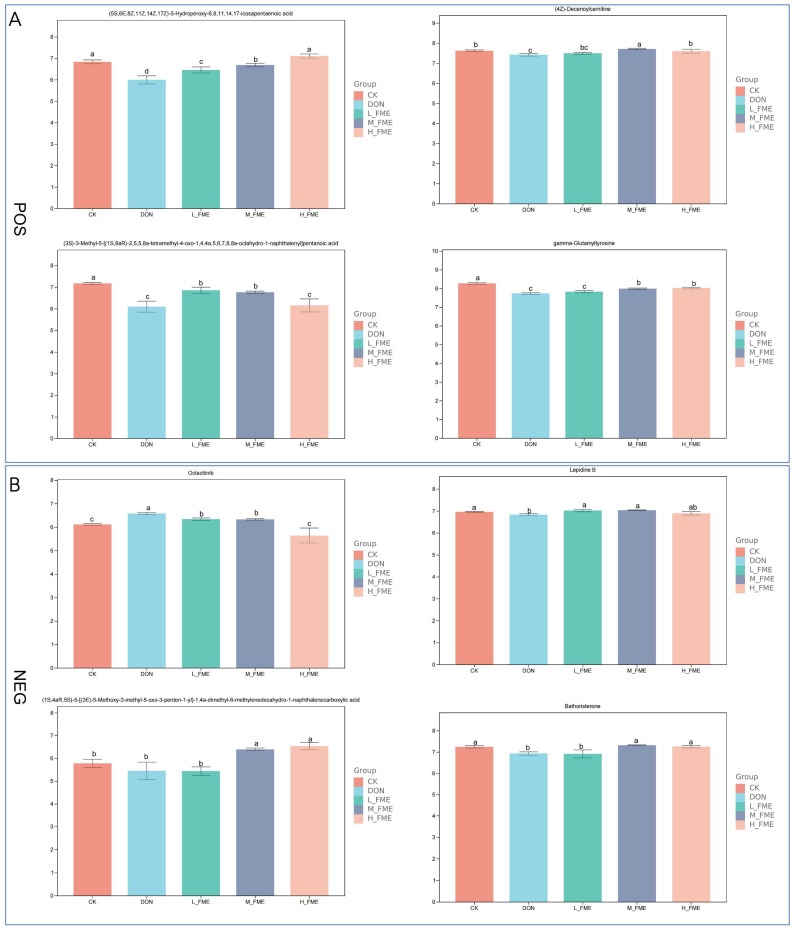
Effects of *Fructus mume* extract (FME) on key hepatic differential metabolites in deoxynivalenol (DON)-exposed mice. (**A**) Key positive metabolites; (**B**) key negative metabolites. CK: control group; DON: DON model group; L-FME: low-dose FME group; M-FME: medium-dose FME group; H-FME: high-dose FME group. Different lowercase letters indicate significant differences among groups (*p* < 0.05). Data are presented as mean ± SEM, *n* = 6.

Key metabolites were further quantified (Figure 9). In positive-ion mode, DON significantly decreased (2E,6E)-2,6-octadienedioic acid, deoxycarnitine, (2S,3R,5S)-5-methyl-6-oxo-3-((2,5-dimethyl-1H-pyrrol-1-yl) methyl) tetrahydro-2H-pyran-2-carboxylic acid, and gamma-glutamyllysine (*p* < 0.01). FME dose-dependently restored these metabolites, with H-FME returning to CK levels (*p* > 0.05).

In negative-ion mode, DON significantly increased clocaprob, D-leucine, and etiocholanone (*p* < 0.01). FME dose-dependently reduced these metabolites. These results indicate that FME alleviates liver injury by enhancing antioxidant and anti-inflammatory metabolism and reducing stress-related metabolites.

### 3.10. Effects of FME on the Gut Microbiota–Liver Metabolite Correlation Network

**Figure 10:**
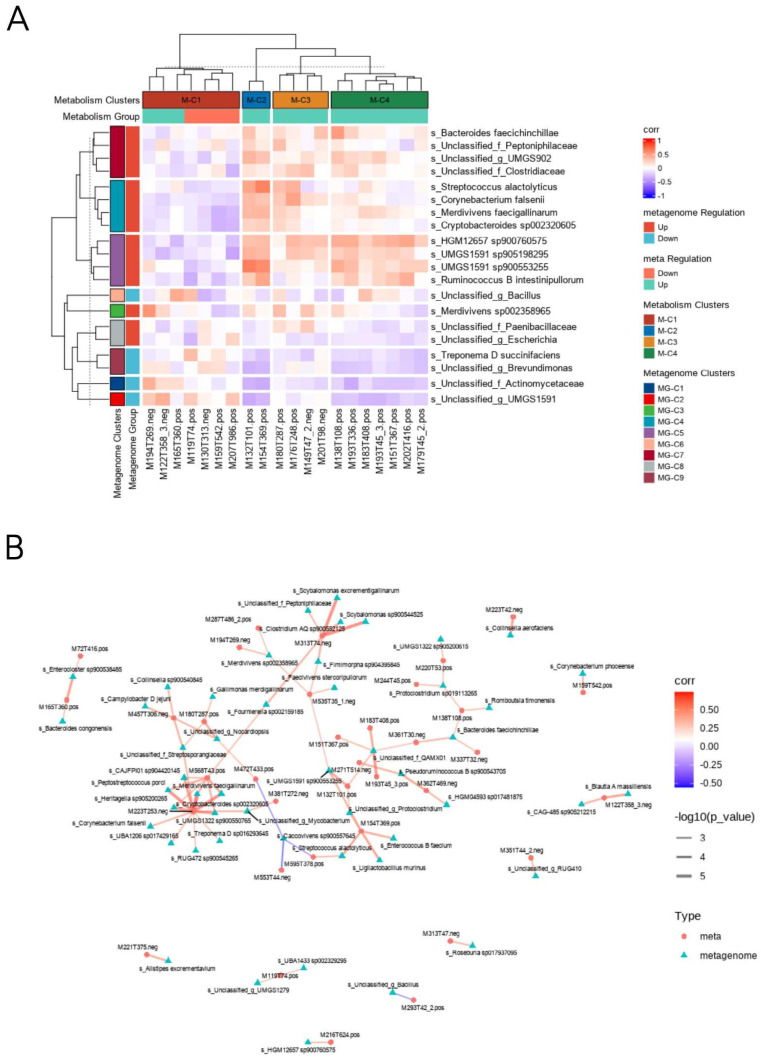
Effects of *Fructus mume* extract (FME) on the gut microbiota–liver metabolite correlation network in deoxynivalenol (DON)-exposed mice. (**A**) Correlation heatmap between gut microbiota and liver metabolites,The different colors on the horizontal axis represent different metabolites, and the different colors on the vertical axis represent different bacterial groups. (**B**) Interaction network of gut microbiota and metabolites. Red dots: metabolites; blue triangles: microbiota. Red line: positive correlation; blue line: negative correlation. Line thickness represents significance (−log_10_(*p*-value)).

Spearman correlation analysis was performed to construct the microbiota–metabolite network (Figure 10). The clustering heatmap (Figure 10A) showed that DON-enriched harmful bacteria were positively correlated with pro-inflammatory metabolites and negatively correlated with protective metabolites. FME increased beneficial bacteria, which were strongly positively correlated with antioxidant and anti-inflammatory metabolites.

The interaction network (Figure 10B) showed that DON formed a strong pro-inflammatory and pro-oxidative correlation network. FME restructured the network by enhancing beneficial microbiota–metabolite interactions and suppressing harmful connections, thereby maintaining homeostasis of the gut–liver axis.

## 4. Discussion

Deoxynivalenol (DON) is one of the most ubiquitous and hazardous mycotoxins contaminating cereals and feed worldwide. Its primary target organ is the intestine, where it induces severe mucosal damage, oxidative stress, inflammatory responses, barrier disruption, and apoptosis [25]. Moreover, DON can disturb gut microbiota and cause secondary liver injury via the gut–liver axis, leading to systemic toxicity [26]. In recent years, natural plant extracts with high safety profiles and antioxidant, anti-inflammatory, and microbiota-regulating activities have become ideal candidates for alleviating DON-induced toxicity. *Fructus mume* (the dried fruit of Prunus mume) is a classic edible and medicinal herb with well-documented astringent, anti-diarrheal, antioxidant, and anti-inflammatory properties. However, its protective effects against DON-induced intestinal and liver injury remain largely unknown. Therefore, in this study, we not only evaluated the protective efficacy of FME against DON-induced intestinal injury using a mouse model, but also investigated its potential molecular mechanisms, with particular emphasis on its anti-inflammatory and antioxidant properties [27].

Body weight change is a direct indicator of overall health status, nutrient utilization, and toxic response in animals. DON exposure has been reported to suppress feed intake and body weight gain by impairing intestinal absorption, inducing inflammatory stress, and disturbing energy metabolism. For example, Zha A et al. found that oral DON exposure caused growth inhibition, metabolic disorders, and histological injury in mice [28]. Similarly, Rajput SA et al. reported that DON-induced intestinal injury was closely associated with reduced growth performance [29]. In line with these findings, the current study confirmed that DON significantly inhibited body weight gain in mice, indicating that the DON model was successfully established. After FME intervention, body weight gain was restored in a dose-dependent manner, and the high-dose FME group reached a body weight level close to that of the control group. This result suggests that FME can alleviate DON-induced growth retardation, which may be related to its ability to improve intestinal morphology, reduce inflammation, and restore metabolic homeostasis.

Histopathological observation is an important method for evaluating structural injury in tissues. In the intestine, villus height reflects absorptive surface area, crypt depth reflects epithelial proliferation and mucosal repair activity, and the villus height/crypt depth ratio indicates the balance between digestive–absorptive capacity and mucosal renewal [30]. Earlier research has shown that DON exposure can impair small-intestinal morphology. Chen J et al. found that feeding nursery pigs with DON-contaminated diets impacted the morphology of the jejunum and ileum, reflected by shifts in villus height, crypt depth, and the villus-to-crypt ratio [31]. Similarly, a recent meta-analysis by Wan S et al. demonstrated that DON exposure significantly impaired production performance and the structure of the small intestine in broilers, including the duodenum, jejunum, and ileum [32]. In addition, Fan et al. showed that DON-induced intestinal toxicity was closely associated with gut microbiota disturbance and intestinal barrier disruption [8]. In this work, DON exposure caused obvious jejunal villus shortening, villus fracture, crypt deepening, and a decreased V/C ratio, indicating severe impairment of intestinal mucosal structure and absorptive function. After FME treatment, villus height increased, crypt depth decreased, and the V/C ratio improved, especially in the high-dose FME group. These findings suggest that FME may restore intestinal absorptive capacity by maintaining villus integrity, reducing excessive crypt hyperplasia, and protecting mucosal barrier function.

The liver is a central organ for metabolism and detoxification, and hepatic histopathological changes are important indicators of DON-induced systemic toxicity. Prior investigations have confirmed that DON exposure can induce hepatic injury, accompanied by pathological alterations, inflammatory responses, immune imbalance, and oxidative stress. Zhou H et al. reported that DON exposure triggered liver inflammation and pathological damage in mice [33], while Feng et al. further showed that DON could induce hepatocyte oxidative stress through the PXR/Malat1-related regulatory axis [4]. In addition, Huang et al. demonstrated that DON caused both intestinal inflammation and liver injury, and that modulation of the gut microbiota could alleviate DON-induced damage along the gut–liver axis [14]. Here, DON exposure resulted in disordered hepatic lobule structure, hepatocyte vacuolar degeneration, and inflammatory cell infiltration, indicating that DON not only damaged the intestine but also induced secondary hepatic injury. FME intervention markedly improved hepatic pathological lesions, and liver morphology in the high-dose FME group was close to that of the control group. These results suggest that FME may protect against DON-induced liver injury, possibly by reducing intestine-derived inflammatory stimulation and restoring hepatic redox and metabolic homeostasis.

Oxidative stress is one of the key mechanisms involved in DON-induced cytotoxicity and organ injury. CAT, SOD, GSH, and T-AOC are major indicators of endogenous antioxidant defense, whereas MDA is a typical lipid peroxidation product reflecting oxidative damage to cellular membranes. Previous studies have shown that DON exposure can disrupt redox homeostasis by promoting ROS accumulation, impairing antioxidant defense systems, and enhancing lipid peroxidation. Chen et al. summarized that DON-induced intestinal toxicity is closely associated with oxidative stress, and that plant-derived polyphenols may counteract this process mainly through activation of antioxidant pathways such as Nrf2 signaling [31]. Zhou H et al. further reported that DON exposure induced liver damage in mice, accompanied by oxidative stress and inflammatory responses [33]. In agreement with these studies, the present results showed that DON significantly decreased hepatic CAT, SOD, GSH, and T-AOC levels, while increasing MDA content, indicating that DON disrupted hepatic redox homeostasis and promoted lipid peroxidation. After FME intervention, antioxidant enzyme activities and total antioxidant capacity were significantly enhanced, whereas MDA content was reduced. Previous studies have demonstrated that *Fructus mume* extract exhibits strong free radical-scavenging activity, including against DPPH, ABTS, hydroxyl, and superoxide radicals [34]. In addition, pharmacological studies on *Prunus mume* have reported that its organic acids, phenolics, and flavonoids are closely related to antioxidant and anti-inflammatory activities [35]. Therefore, the protective effect of FME against DON-induced liver injury may be partly attributed to its ability to reduce lipid peroxidation, enhance endogenous antioxidant defense, and restore hepatic redox balance.

Inflammation is another important pathological process involved in DON-induced tissue injury. NF-κB is a classical inflammatory signaling molecule that regulates the transcription of multiple pro-inflammatory mediators, including IL-6, IL-1β, TNF-α, and IFN-γ. IL-10 is an important anti-inflammatory cytokine that limits excessive inflammatory responses, whereas IgG reflects humoral immune status. Previous studies have shown that DON-induced cytotoxicity is closely associated with activation of inflammatory signaling pathways. Krishnaswamy et al. reported that DON exposure promoted NF-κB-related inflammatory responses in intestinal epithelial cells [36]. More recently, Chen et al. showed that chlorogenic acid attenuated DON-induced apoptosis and pyroptosis by activating the Nrf2/HO-1 pathway and inhibiting MAPK/NF-κB/NLRP3 signaling [37], while Xue et al. further demonstrated that DON-induced IPEC-J2 cell pyroptosis was associated with activation of the NF-κB/NLRP3/caspase-1 pathway [38]. In the present study, DON significantly upregulated the expression of *NF-κB*, *IL-6*, *IL-1β*, *TNF-α*, and *IFN-γ* in liver and jejunum tissues, while reducing IL-10 and serum IgG levels. These data show that DON induced both local intestinal inflammation and systemic immune imbalance. FME treatment dose-dependently suppressed NF-κB activation, lowered pro-inflammatory cytokine expression, and elevated IL-10 and IgG levels. In agreement with prior reports, Fructus mume extracts possess anti-inflammatory activity and can alleviate intestinal inflammatory diseases, including experimental colitis and Crohn’s disease-like injury [18,27]. Therefore, FME may relieve DON-triggered inflammatory injury via two mechanisms: inhibiting NF-κB-mediated signaling and rebalancing pro- and anti-inflammatory responses.

Epithelial cells and tight junction proteins mainly maintain the intestinal barrier. Among these proteins, ZO-1, Occludin, and Claudin-1 are critical for regulating paracellular permeability and preserving barrier integrity. Prior research has shown that DON exposure damages the intestinal epithelial barrier by disrupting tight junction architecture and lowering tight junction protein levels. Pinton et al. reported that DON reduced intestinal barrier function and decreased claudin expression, providing early evidence of DON’s barrier-disrupting effect [39]. More recently, Alberge et al. showed that DON induced epithelial barrier dysfunction in cell monolayers originating from pig jejunum organoids [40], while Hu et al. demonstrated that activation of AHR alleviated DON-induced impairment of porcine intestinal epithelial barrier function [41]. In addition, Miao et al. reported that DON induced intestinal epithelial barrier damage via RhoA/ROCK pathway-mediated apoptosis and F-actin-associated tight junction disruption [42]. In the present study, DON significantly reduced the mRNA and protein expression of *ZO-1*, *Occludin*, and *Claudin-1* in jejunal tissue, confirming that DON disrupted the intestinal physical barrier. After FME treatment, the expression and localization of these tight junction proteins were restored in a dose-dependent manner. Collectively, these results demonstrate that FME can repair DON-induced barrier dysfunction by enhancing tight junction protein expression, thereby diminishing intestinal permeability, preventing toxins from translocating, and interrupting the amplification of inflammation through the gut–liver axis.

Excessive apoptosis of intestinal epithelial cells is an important cause of mucosal barrier destruction. TUNEL staining directly reflects apoptotic cells, while Tradd, Caspase-3, Caspase-7, Caspase-8, and BAX are closely associated with apoptotic activation. Bcl-2 is an anti-apoptotic molecule that helps maintain cell survival. Earlier research has shown that DON-induced intestinal epithelial injury is closely related to oxidative stress and apoptosis. Krishnaswamy et al. reported that DON induced oxidative stress and apoptosis in HT-29 intestinal epithelial cells, accompanied by changes in NF-κB-related signaling [36]. In addition, Chen et al. showed that chlorogenic acid attenuated DON-induced apoptosis and pyroptosis by activating Nrf2/HO-1 and inhibiting MAPK/NF-κB/NLRP3 signaling [31]. In the present study, DON markedly increased TUNEL-positive cells in the jejunum, upregulated the expression of *Tradd*, *Caspase-3*, *Caspase-7*, *Caspase-8*, and *BAX*, and downregulated *Bcl-2* expression, indicating that DON induced severe intestinal epithelial apoptosis. FME intervention significantly reduced apoptotic cells, suppressed pro-apoptotic gene expression, and increased *Bcl-2* expression. These findings suggest that FME protects intestinal epithelial integrity not only by promoting tight junction repair but also by inhibiting excessive epithelial apoptosis. Therefore, the anti-apoptotic effect of FME may be another key mechanism underlying its protection against DON-induced intestinal barrier injury.

Gut microbiota diversity and composition are important indicators of intestinal microecological homeostasis. Chao1 and ACE reflect microbial richness, and Shannon and Simpson indices reflect microbial diversity and evenness, while PCoA and UMAP analyses show differences in microbial community structure. Previous research has demonstrated that DON exposure is strongly linked to intestinal microbiota disturbance and intestinal injury. Bai et al. demonstrated that the gut microbiota participated in DON-induced anorexia and that Lactobacillus rhamnosus GG alleviated DON-induced adverse effects through microbiota modulation [43]. Zhai et al. reported that DON exposure could damage the intestinal barrier and disrupt the gut microbiota, thereby aggravating intestinal dysfunction [44]. However, Liao et al. reported that DON-induced colon damage in mice could occur independently of the gut microbiota, suggesting that the causal relationship between DON-induced intestinal injury and microbiota dysbiosis should be interpreted with caution [45]. Consistent with microbiota disturbance observed in previous studies, our study showed that DON significantly decreased α-diversity and caused a clear separation of gut microbial structure from that of the control group. FME intervention restored microbial diversity and shifted the microbial community toward the control pattern in a dose-dependent manner. At the taxonomic level, FME increased the abundance of Firmicutes, Actinobacteriota, Lachnospiraceae, Oscillospiraceae, Clostridium, Oscillibacter, and Alistipes, while reducing the abundance of potentially harmful bacteria. Many beneficial gut bacteria are associated with short-chain fatty acid production, anti-inflammatory regulation, epithelial barrier maintenance, and host energy metabolism [14,46]. Therefore, FME may alleviate DON-induced intestinal injury by rebuilding a healthier intestinal microecological environment, although further causal validation using fecal microbiota transplantation or germ-free animal models is still needed.

Hepatic metabolomics provides insight into systemic metabolic changes induced by DON and the regulatory effects of FME on metabolism. Previous studies have shown that DON can disturb lipid metabolism, amino acid metabolism, bile acid metabolism, and oxidative stress-related pathways, ultimately impairing liver function and energy homeostasis [44]. In the present study, DON exposure caused a clear separation of hepatic metabolic profiles from the control group, with marked changes in lipid-like molecules, organic acids, amino acid-related metabolites, and antioxidant-associated metabolites. FME treatment gradually shifted the hepatic metabolic profile toward that of the control group and regulated several key differential metabolites. These results suggest that FME not only reduces histological and inflammatory damage but also improves DON-induced metabolic reprogramming. In particular, the regulation of metabolites related to antioxidant defense, lipid metabolism, and amino acid metabolism may contribute to the restoration of hepatic function.

The gut–liver axis provides an important theoretical basis for explaining the simultaneous intestinal and hepatic protection observed in this study. Gut microbiota-derived metabolites and intestinal inflammatory mediators can enter the liver through the portal circulation, thereby affecting hepatic immunity, oxidative stress, and metabolic processes. Recent studies have provided direct evidence that DON-induced intestinal dysbiosis and barrier disruption are closely associated with hepatic injury. More directly, Jin et al. demonstrated, using oral DON exposure and fecal microbiota transplantation experiments, that gut microbiota dysbiosis could transmit DON toxicity to the liver by activating the TLR4/MyD88/NF-κB inflammatory pathway [47]. In addition, Wen et al. showed that DON exposure caused cecal microbiota dysbiosis, intestinal barrier disruption, liver damage, and lipid metabolic disorders via the microbiota–gut–liver axis [48]. In our study, correlation analysis showed that DON-enriched harmful bacteria were positively associated with pro-inflammatory or pro-oxidative metabolites, whereas FME-enriched beneficial bacteria were positively associated with protective metabolites. This result suggests that FME may reconstruct the gut microbiota–liver metabolite interaction network and reduce the transmission of intestinal injury signals to the liver. Although correlation analysis cannot directly prove causality, these findings provide important evidence that the protective effect of FME may be closely related to the regulation of gut–liver axis homeostasis.

In conclusion, the present study demonstrates that FME effectively alleviates DON-induced intestinal and liver injury in mice. Its protective mechanisms may involve enhancing antioxidant capacity, inhibiting NF-κB-mediated inflammation, restoring tight junction proteins, suppressing intestinal epithelial apoptosis, reconstructing the gut microbiota, and regulating hepatic metabolic homeostasis. These findings provide experimental evidence for the potential application of FME as a natural protective agent against DON toxicity and offer a theoretical basis for further development of *Fructus mume*-derived functional products.

## 5. Conclusions

The results of this study suggest that FME may protect mice against DON-induced toxicity. The underlying mechanisms involve enhancing antioxidant capacity and reducing oxidative stress; inhibiting NF-κB-mediated inflammatory responses and restoring immune function; upregulating tight junction proteins and suppressing apoptosis to repair the intestinal barrier; regulating gut microbiota composition and restoring microbial diversity; and normalizing hepatic metabolism and maintaining gut–liver axis homeostasis. This study provides scientific evidence for FME as a promising natural intervention to mitigate DON toxicity in food and feed. Future studies will focus on isolating and identifying key active components of FME and exploring their precise molecular targets.

## Figures and Tables

**Table 1:** mRNA sequence.

Gene	Primer Sequence (5′-3′)	Product Length (bp)
*Caspase-3*	ATGGGAGCAAGTCAGTGGAC	146 bp
	GTCCACATCCGTACCAGAGC	
*B* *AX*	AGATGAACTGGACAGCAATATGG	199 bp
	GATCAGCTCGGGCACTTTAG	
*Bcl-2*	AGCCTGAGAGCAACCCAATG	165 bp
	GACGGTAGCGACGAGAGAAG	
*Tradd*	GGTGGAGCCATACAGGTAGC	129 bp
	CAGTGGCCGGTTCACTACG	
*Caspase* *-* *7*	ACTTCGACAAAGCGACAGGT	116 bp
	AGAGCAGTCATTGTGGACGG	
*Caspase* *-* *8*	TGCTTGGACTACATCCCACAC	171 bp
	GTTGCAGTCTAGGAAGTTGACC	
*ZO-1*	GAGCAAGCCTTCTGCACATC	111 bp
	TCGGGTTTTCCCTTTGAAGAGT	
*Occludin*	ATGGCTGCTGCTGATGAATA	111 bp
	CTTGATGTGCGATAATTTGCTCTT	
*Claudin-1*	TTCTCTGGGATGGATCGGCT	106 bp
	TCCCTCGTAGATGGCCTGAG	
*TNF-α*	CAGGCGGTGCCTATGTCTC	276 bp
	CGATCACCCCGAAGTTCAGTAG	
*IFN-γ*	AACCTCACCTACAGGGCGGACTTCA	148 bp
	TCCCACGTCAATCTTTCCTCTTGCTTT	
*IL-6*	CATGTTCTCTGGGAAATCGTG	232 bp
	TCCAGTTTGGTAGCATCCATC	
*IL-1β*	CGGCGAGATCAGAACCTACAAC	145 bp
	GGCACTGTCACACTGGTCACTC	
*NF-κ* *B*	GCTTCCGGGAGCCTCTAGTG	83 bp
	TAACCTTTGCAGGCCCCACA	
*IL-10*	ATGCAGGACTTTAAGGGTTACTTG	253 bp
	AGACACCTTGGTCTTGGAGCTTA	
*β-actin*	CACTGCCGCATCCTCTTCCTCCC	150 bp
	CAATAGTGATGACCTGGCCGT	

**Table 2:** Compounds identified in *Fructus mume* extract.

Components	Retention Time/min	Mode of Acquisition	Parent Ions (*m*/*z*)	Fragment Ions (*m*/*z*)	Peak Area
Ononin	5.51	[M+H]+	430.9	269.1	1,002,226.57499334
Citric Acid	1.49	[M-H]-	191	86.9	10,721,017.6624854
Abscisic Acid	5.8	[M-H]-	263.1	153.1	1,074,264.12776566
Biochanin A	6.56	[M-H]-	283.1	267.9	1,364,991.63520378
Butin	5.51	[M-H]-	271	135	1,403,733.58469897
(E)-Ferulic Acid	5.31	[M-H]-	193	134.1	1,597,106.18575201
Benzoic Acid	5.59	[M-H]-	120.9	77.1	2,173,375.81283318
Trigonelline	0.78	[M+H]+	138.2	94	22,366.836412096
Liquiritigenin	5.75	[M-H]-	255.2	119	24,266,123.9189028
2-Hydroxyphenylacetic Acid	5.14	[M-H]-	151	107	244,837.041587098
Pinocembrin	6.5	[M-H]-	255	151	24,927.3491764148
Calystegine A3	4.46	[M-H]-	158	116.1	25,539.649580139
Loganic Acid	4.37	[M-H]-	375.2	213	278,280.309291202
Ferulic Acid	5.33	[M-H]-	193	134	2,798,567.13482947
Nicotiflorin	5.13	[M+H]+	595	287.2	284,707.330885611
Mucic Acid	0.73	[M-H]-	209	85	348,934.732532148
Gentiopicroside	4.81	[M+Na]+	379.1	109	35,400.7662082605
P-Hydroxybenzaldehyde	5.11	[M-H]-	120.9	92	383,028.696299533
Rutin	5.02	[M+H]+	611.1	303	38,533.3738421788
Perillyl Alcohol	5.25	[M-H]-	151.1	107.2	423,921.474118324
P-Hydroxycinnamic Acid	5.21	[M-H]-	162.9	119	4,281,600.24361435
Estragole	7.64	[M+H]+	149.2	93.1	459,289.224744001
Alpha-ketoglutaric acid	0.83	[M+FA-H]-	191	111	475,331.541473389
Isoquercitrin	5.12	[M-H]-	463	300	490,963.768225708
Stachyose	0.76	[M+H-H_2_O]+	649.2	325.1	49,634.8113479487
Calycosin	5.78	[M+H]+	285.3	213.2	50,501.1580044301
Eriodictyol	5.75	[M-H]-	287.1	151	656,807.337998323
Sclareol	7.15	[M+HCOOH]-	353.3	353.3	6,597,586.38126898
Crocetin-1	6.63	[M-H]-	327.1	283.2	663,653.433283206
Butin-1	5.49	[M-H]-	271	135	681,785.210967685
Quercitrin	5.29	[M-H]-	447.1	300.9	70,402.5759415318
D-Gluconic Acid	0.74	[M-H]-	195	129	7,237,097.46507822
Formononetin	6.25	[M+H]+	269.3	197.1	758,009.879708355
Isoformononetin	6.25	[M+H]+	269.3	226.1	775,146.611820029
(E)-M-Coumaric Acid	5.36	[M-H]-	162.9	119.1	800,834.478618797
(+)-Afzelechin	0.81	[M+H]+	275	197	83,367.6682433016
(R)-(+)-Citronellal	4.15	[M-H]-	153.2	109	8,588,629.46013503
Naringin	5.24	[M-H]-	579.1	271	97,754.5734988252

*m*/*z*: first-order mass spectrometry.

## Data Availability

The data presented in this study are openly available in [Mendeley Data] [Reserved DOI: 10.17632/h734wgnzfb.1].
